# Vapor Pressure versus Temperature Relations of Common Elements

**DOI:** 10.3390/ma16010050

**Published:** 2022-12-21

**Authors:** B. Mondal, T. Mukherjee, N. W. Finch, A. Saha, M. Z. Gao, T. A. Palmer, T. DebRoy

**Affiliations:** 1 Department of Materials Science and Engineering, The Pennsylvania State University, University Park, PA 16802, USA; 2 Department of Engineering Science and Mechanics, The Pennsylvania State University, University Park, PA 16802, USA

**Keywords:** vapor pressure, elevated temperature, optimization, differential evolution, evaporation

## Abstract

The vapor pressure values of common elements are available in the literature over a limited temperature range and the accuracy and reliability of the reported data are not generally available. We evaluate the reliability and uncertainty of the available vapor pressure versus temperature data of fifty common pure elements and recommend vapor pressure versus temperature relations. By synthesizing the vapor pressure values from measurements reported in the literature with the values computed using the Clausius Clapeyron relation beyond the boiling point, we extend the vapor pressure range from 10^−8^ atm to 10 atm. We use a genetic algorithm to optimize the fitting of the vapor pressure data as a function of temperature over the extended vapor pressure range for each element. The recommended vapor pressure values are compared with the corresponding literature values to examine the reliability of the recommended values.

## 1. Introduction

The vapor pressures of elements at various temperatures are important for a wide range of scientific and engineering calculations [1,2,3,4,5,6]. Vapor pressure data are important for many metal processing operations and properties of many alloys. They are needed to predict the loss of alloying elements due to vaporization during additive manufacturing and fusion welding and for the deposition of various thin films of commercial interest [1,2,3,4,5,6]. In the keyhole mode welding and additive manufacturing processes, the relationship between temperature and vapor pressure is a requisite to predict the shape, size, and stability of the keyhole [7]. Similarly, in the pyrometallurgical production of metals, vapor pressure and the rates of evaporation of zinc and cadmium are used in the final refining steps of their extraction [8,9]. Accurate knowledge of the vapor pressure is necessary to have a vapor coating of elements [10]. In high-pressure systems such as nuclear reactors, the choice of coolants like liquid sodium or alloys of sodium-potassium and lead-bismuth is affected by their vapor pressures [11]. Therefore, an accurate database of vapor pressure for elements is needed for different scientific and technological applications.

Despite the importance of vapor pressure data, work on the vapor pressure of elements has not advanced much since the 1980s when Hultgren compiled the vapor pressure data of several elements [12]. These vapor pressure data at various temperatures were fitted by Alcock et al. [13] and Gale et al. [14], using linear regression to provide relations between vapor pressure and temperature. However, for most elements, the resulting fitted equations are valid for a narrow temperature range much below the boiling point of the liquid. For example, for Vanadium with a boiling point of 3680 K, the vapor pressure equation from Smithells Metals Handbook is only valid till 2175 K leaving a temperature range of 1505 K below the boiling point with no vapor pressure-temperature data. The second major issue is that these sources provide multiple equations to represent the change in vapor pressure for different temperature ranges. For example, while Gale et al. [14] uses two equations for several elements, Alcock et al. [13] uses two equations for each element. Finally, for several elements, the temperature which corresponds to 1 atm pressure does not match the boiling point of the elements. For example, in the case of calcium, the predicted boiling point using the Gale et al. [14] relation differs from the literature boiling point by 100 K. What is needed and currently not available are vapor pressure values of elements over a wide range of temperatures and the reliability and uncertainty of the data.

We seek to develop a single vapor pressure-temperature relation valid for a wider temperature, i.e., up to a maximum pressure of 10 atm which can also correctly predict the boiling point of the element. This work uses the experimental data reported in the literature and synthesized data using Clausius Clapeyron relation to represent vapor pressure over a large range of temperatures for fifty elements. For each of the fifty elements, the resulting vapor pressure versus temperature data was fitted into an equation. The fitting of the vapor pressure versus temperature data was optimized using a genetic algorithm (GA) and the accuracy of the fitting was evaluated. Finally, the reliability of the recommended pressure versus temperature relation was examined by comparing the recommended values with the corresponding values reported in the literature.

## 2. Methodology

The experimental data of vapor pressure versus temperature were collected from the literature and where data were not available, the Clausius Clapeyron thermodynamic relation was used to fill in the gaps in the available data. The resulting data were fitted to an equation for each element. The data fitting was optimized using a differential evolution (DE) algorithm [15,16,17]. The methods of data collection and data fitting optimization are discussed below.

### 2.1. Data Collection

We collected the vapor pressure data ranging from 10^−8^ atm (1.013 × 10^−3^ Pa) to 10 atm (1.013 × 10^6^ Pa). The data at low pressure and temperatures below the boiling point are available in the literature [12]. These data were collected for all fifty elements [12]. The lowest pressures for which data was collected [12] is 10^−8^ atm because this pressure corresponds to the ultra-high vacuum achieved by most commercial equipment [18]. At high temperatures, vapor pressure data are not available. We assumed that the vapor behaves as an ideal gas and estimated the vapor pressure using the Clausius-Clapeyron equation [19] as,

(1) $$\ln\left(\frac{p_{1}}{p_{2}}\right)=-\frac{\Delta H_{vap}}{R}\left(\frac{1}{T_{2}}-\frac{1}{T_{1}}\right)\;\;$$

where $\Delta H_{vap}$ is the enthalpy of vaporization in J/mol and is assumed to be independent of temperature. $P_{1}$ and $P_{2}$ are pressures in atm, at temperatures $T_{1}$ and $T_{2}$ in Kelvin, respectively. Using $P_{1}$ as 1 atm and $T_{1}$ as the normal boiling point of an element, we calculated the pressure $P_{2}$ at temperature-$T_{2}$, the temperature of interest. The symbol *R* represents the gas constant (8.314 J/mol-K). Thus, vapor pressure data at temperatures above the boiling point were generated. Table 1 lists the boiling point and the enthalpy of vaporization of all fifty elements [20,21]. The vapor pressures were calculated using the Clausius-Clapeyron relationship up to 10 atm. The upper limit of 10 atmospheres is considered to limit the uncertainty of the predicted values.

The collected vapor pressure data ranging from 10^−8^ atm to 10 atm were used as the input data for a genetic algorithm to determine the coefficients A, B, C, and D of an equation of the following form [13,14],

(2) $$\log\left(P\right)=-\frac{A}{T}+B+C\;\cdot \log\left(T\right)+10^{-3}\cdot D\cdot T\;\;$$

Here, *T* has units of Kelvin, and *P* is pressure in atmospheres. Genetic Algorithm optimizes the values of the four coefficients *A*, *B*, *C*, and *D* to achieve the best data fitting as discussed below.

### 2.2. Data Fitting Optimization Using the Differential Evolution Genetic Algorithm

The genetic algorithm (GA) used a differential evolution (DE) method that has been demonstrated in many scientific and technological problems like the determination of the ground state of Si-H crystals [16] and the determination of earthquake hypocenter [17].

**Figure 1:**
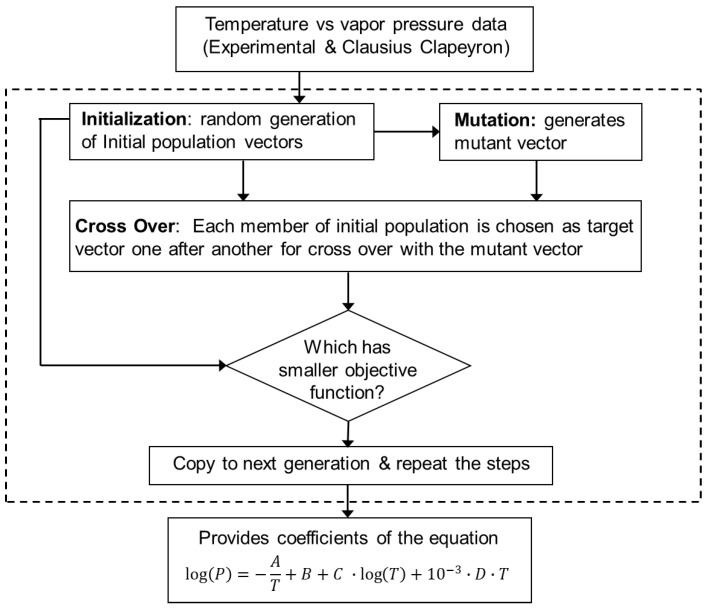
The overall structure of this work. Data collected from experimental work and synthesized using the Clausius Clapeyron equation is fed to a differential evolution genetic algorithm (GA) to provide the coefficients *A*, *B*, *C*, and *D* of the vapor pressure relation. The dotted box indicates the GA algorithm.

Figure 1 shows schematically the various steps of the DE optimization algorithm for each element. First, DE randomly selected an initial population of *A*, *B*, *C* and *D*. Each of the population contained ten vectors to improve the accuracy of the data fitting. Each vector had four elements corresponding to the four coefficients *A*, *B*, *C*, and *D* in Equation (2). Next, additional vectors were generated through the process of mutation where an additional mutant vector can be expressed as,

(3) $$V{\left(i\right)}_{mutant}=V_{j}\left(i\right)+mf\cdot \left(V_{k}\left(i\right)-V_{l}\left(i\right)\right)\;\;$$

where $V_{j},\;\;V_{k},$ and $V_{l}$ are random initial population vectors, ‘mf’ is the mutation factor that controls the evolution of the population. The index ‘i’ corresponds to the elements in the vector (coefficients *A*, *B*, *C*, and *D*).

After the mutation, the mutant vectors were combined with the initial population vector to generate a trial vector. This process is called cross-over. The trial vector was tested against the initial population vector using an objective function represented as,

(4) $$f=\sum_{1}^{n}{\left(logP-\left(-\frac{A}{T}+B+C\;logT+10^{-3}DT\right)\right)}^{2}\;$$

where f is the sum of the squared difference between vapor pressure (*P*), and the values calculated by the coefficients from the differential evolution algorithm, and ‘*n*’ is the number of data points. ‘*f*’ also indicates the fitness value for each population. For the comparison of the initial population vector against the trial vector, the vector with the lowest value of f is kept for the next generation. This comparison is repeated for each vector of the population. When the comparison for all population vectors in a generation was concluded, the process was repeated until the total number of generations was completed. The total number of generations was chosen to be 500,000. The above process was repeated for each of the fifty elements to obtain the coefficients *A*, *B*, *C*, and *D*. The calculation was done using an in-house FORTRAN code compiled using the Intel^®^ Fortran Compiler, ifort version 2021.7.0.

## 3. Results and Discussion

### 3.1. Improved Vapor Pressure Relation

**Figure 2:**
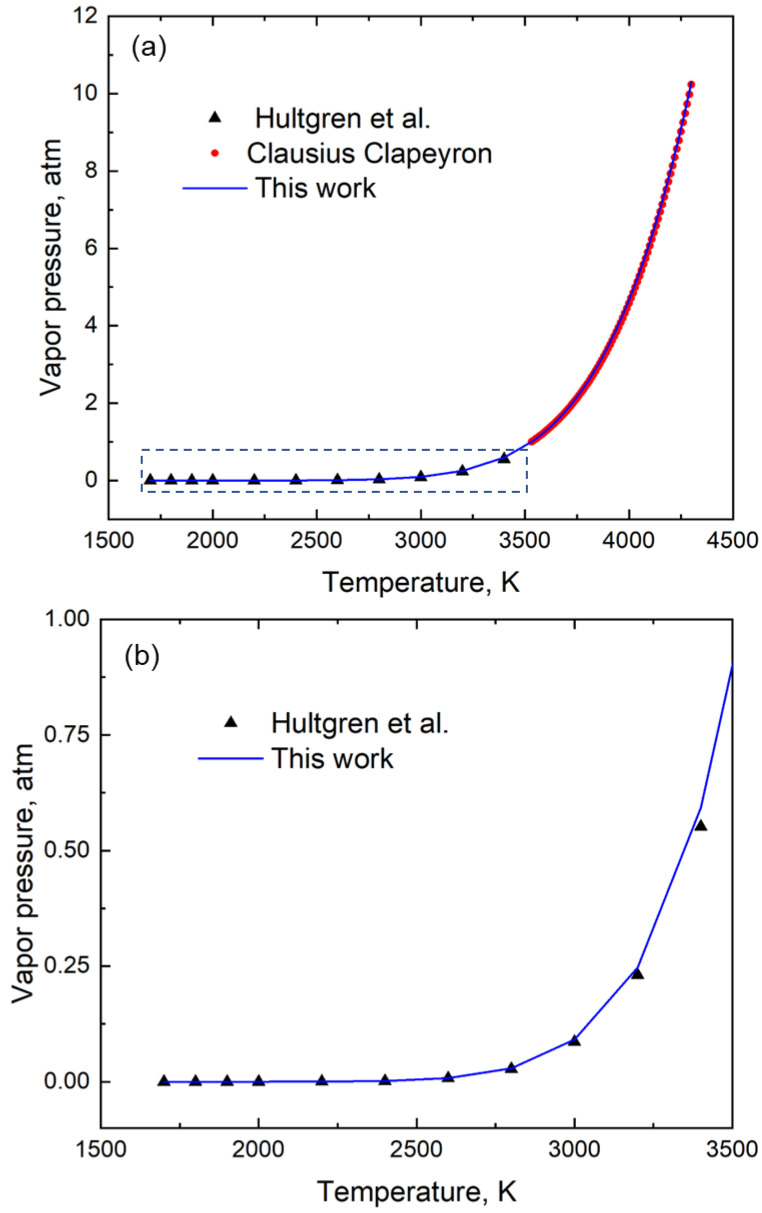
(**a**) A plot of vapor pressure with temperature for Silicon (Si). The coefficients *A* = 17,250, *B* = −15.97, *C* = 6.403 and *D* = −0.5281 shown in Table 2 are used in Equation (2) to generate the blue curve in this plot. The region marked by the rectangle is shown separately in 2(b). (**b**) Enlarged section of the vapor pressure data between 1500 K and 3500 K shows a good fit with the equation. The experimental data from Hultgren et al. [12] and the fitting results between 1700 K and 3400 K are tabulated in the Appendix A.

Table 2 reports the coefficients *A*, *B*, *C*, and *D* of the vapor pressure-temperature relation (shown in Equation (2)) for fifty elements. These coefficients were derived using the genetic algorithm method of optimization as explained earlier. Figure 2 shows an example of the optimization of the fitting using the element silicon. In this figure, the blue line represents the vapor pressure-temperature relation between 1700 K and 4300 K. This blue line is generated from the vapor pressure versus temperature data using its coefficients *A*, *B*, *C*, and *D* (Table 2) in Equation 2 obtained using a genetic algorithm. The black triangles represent the experimental vapor pressure data between the temperature of 1700 K and 3400 K taken from Hultgren’s handbook [12]. The vapor pressure data synthesized using the Clausius Clapeyron equation and the enthalpy of vaporization and boiling point information [20] is shown by the red circles in the plot (Figure 2). The first red circle represents the boiling point (3533 K) corresponding to 1 atm pressure and the last circle corresponds to a pressure near 10 atm, i.e., a temperature of 4300 K. This combined experimental and synthesized vapor pressure data of Si represented by the black triangles and red circles were used in GA to calculate the coefficients of the equation. The experimental data from Hultgren et al. [12] and the corresponding fitted results using the coefficients of GA is provided in Table A1 of Appendix A. Using the coefficients for element Si, the temperature corresponding to 1 atm pressure is predicted to be the boiling point of the element which is calculated to be 3533 K. The boiling point of Si as reported in the literature [20] is 3533 K. We thus show that our single vapor pressure-temperature relation is valid for a wide temperature while also correctly predicting the boiling point of the element. 

To represent the utility of the relation for the entire range of pressure, a root mean square error (*RMSE*) is provided along with the coefficients in Table 2. *RMSE* is calculated based on the difference between the vapor pressure versus temperature relation using the optimized coefficients and the pressure that was calculated in data collection stage is represented as

(5) $$RMSE=\sqrt{\frac{\sum_{1}^{n}{\left(P_{lit}-P_{GA}\right)}^{2}}{n}}\;\;\;\;$$

where $P_{lit}$ corresponds to the pressure obtained from literature or using Clausius Clapeyron relation. $P_{GA}$ is the pressure calculated using the coefficients provided by GA and *n* is the number of data points. *RMSE* for the fifty elements are provided in Table 2.

**Figure 3:**
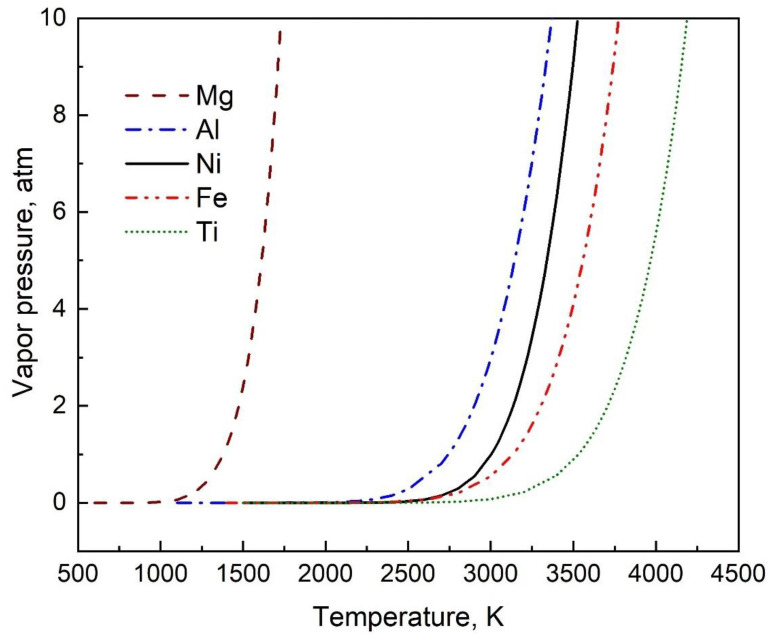
The variation of vapor pressure with temperature for five commonly used elements of Mg, Al, Ni, Fe, and Ti using the coefficients generated from this study (Table 2) in Equation (2).

The variation of vapor pressure with temperature for five commonly used elements of Mg, Al, Ni, Fe, and Ti are obtained using the coefficients generated from this study (Table 2) and is shown in Figure 3. 

**Figure 4:**
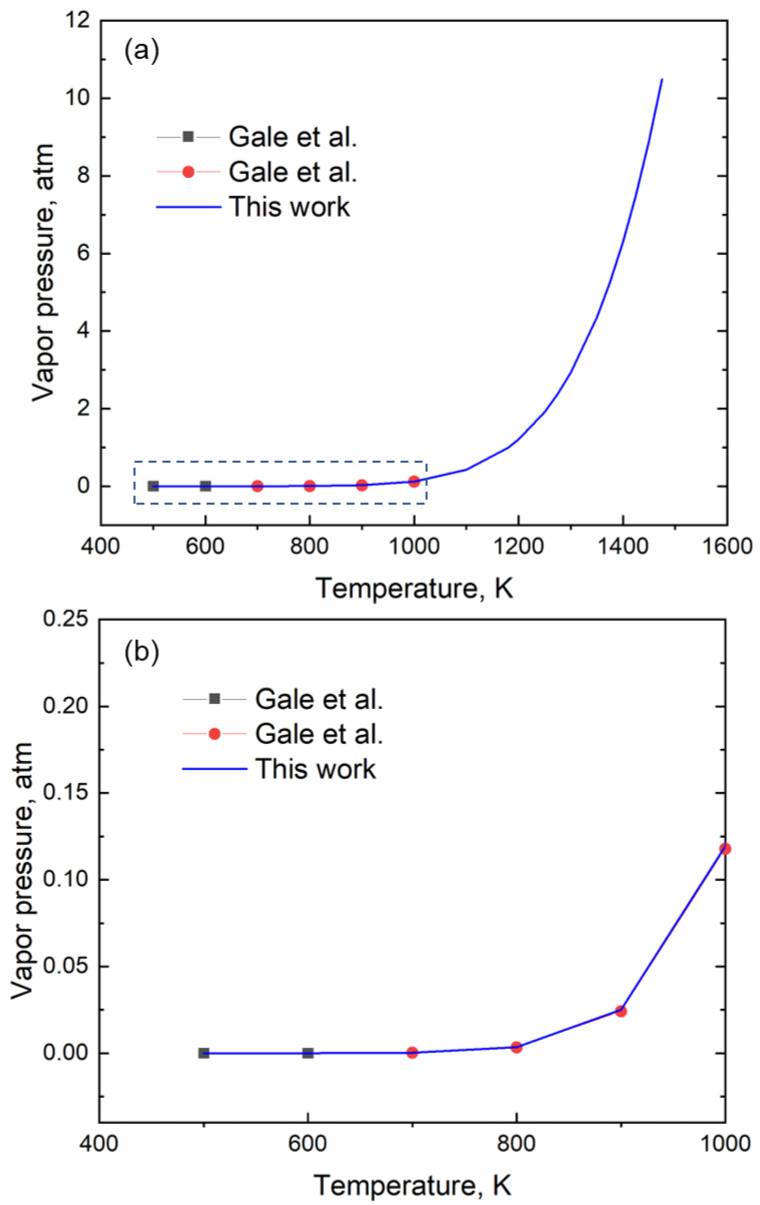
(**a**) A plot of the vapor pressure data of Zn using data from the handbook and the coefficients generated in this study. While Gale et al. [14] provides two different relations denoted by the black squares (between the temperature of 473 K to 692.5 K) and red circles (temperature of 692. K to 1000 K), our work represents the variation in vapor pressure data using a single relation. (**b**) The enlarged section of the low-temperature vapor pressure data between 400 K and 1000 K shows a good fit with the equation.

We show that a single relation is sufficient to represent the entire range of vapor pressure even for the elements for which two or more relations were needed. For example, Gale et al. [14] used two equations to define the vapor pressure of Zn between 500 and 1000 K, where one equation was for 473 K to 692.5 K and the other was for 692.5 K to 1000 K. These two relations are represented by the black squares and red circles in Figure 4, respectively. Here, we provide a single equation, represented by the blue line, that can be used to describe the vapor pressure over the entire temperature range of 500 to 1475 K accurately. Thus, the coefficients for Zn derived from GA are valid from 500 K to 1475 K and provide vapor pressure with a mean absolute error of 4.44 × 10^−4^ atm (Figure 4). 

**Figure 5:**
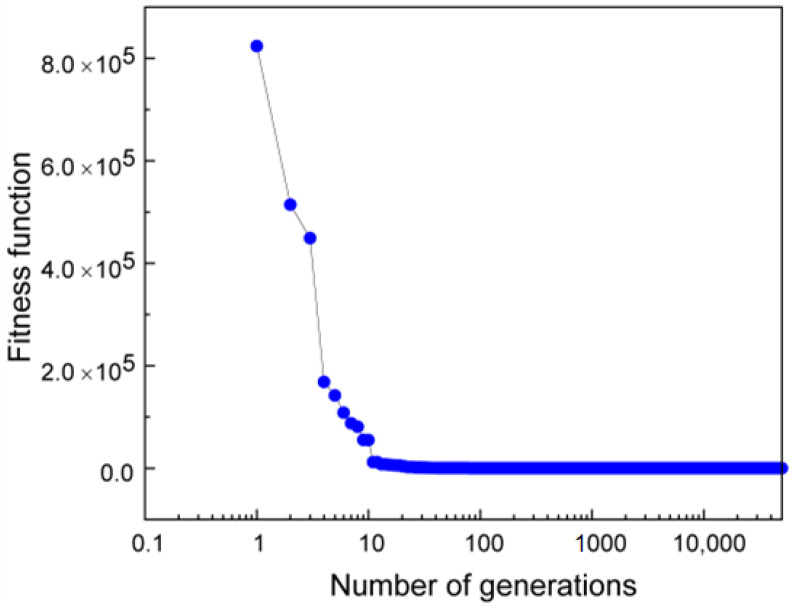
A plot showing the decrease in fitness function with the number of generations for Silicon (Si).

The average fitness error (F) that represents the soundness of the data fitting by GA for each generation is calculated as

(6) $$F=\frac{1}{N}\;\overset{N}{\underset{1}{\sum }}f\;$$

where ‘*N*’ is the number of populations and ‘*f*’ is calculated using Equation (4). A plot of the average fitness error as a function of number of generations for Si is shown in Figure 5. Fitness error decreases rapidly from 8 × 10^5^ for the initial population to 922, 16, 0.46, and 0.01 in the 30th, 100th 1000th, and 10,000th generation, respectively, and finally to 0.002 at the end of the 50,000th generation. This indicates that the GA converges rapidly and provides a very good fitting indicated by the low fitness error. The relations provided by GA are tested using independent experimental data as discussed below.

### 3.2. Verification with Data

**Figure 6:**
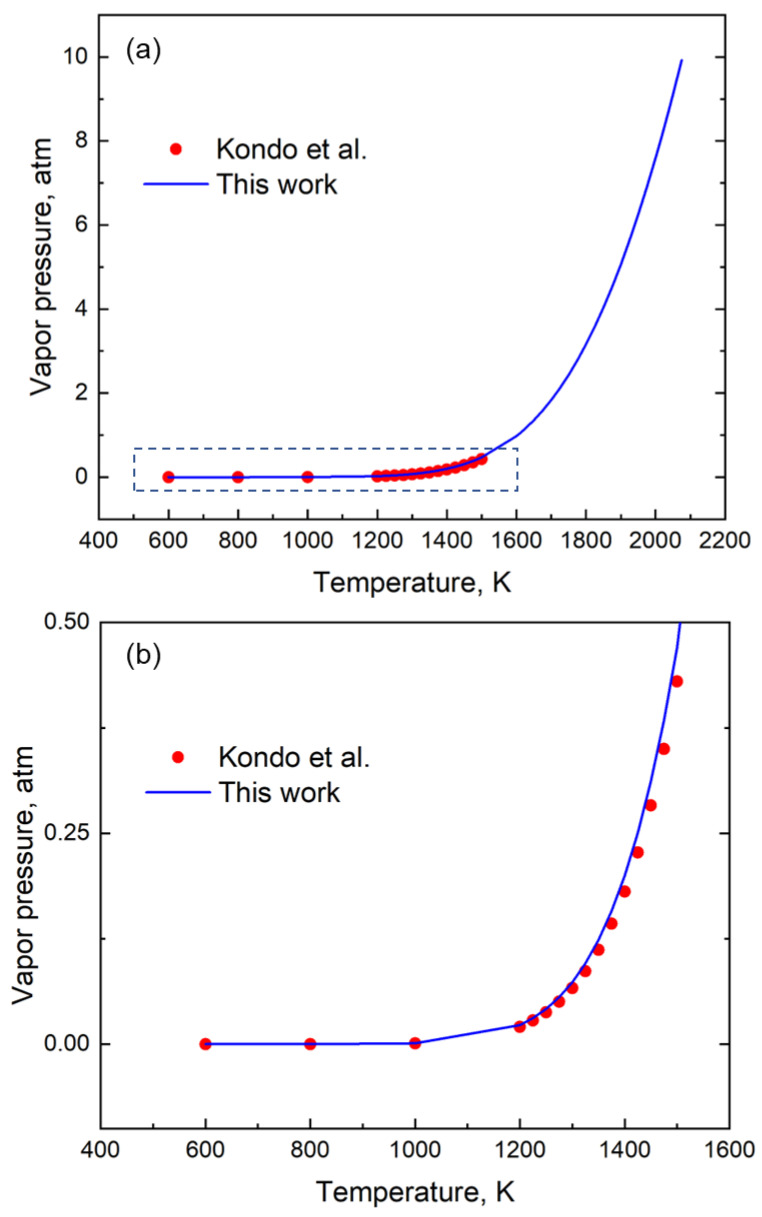
(**a**) A Comparison of the vapor pressure of Li using coefficients generated using our method (GA) and that of Kondo et al. [22]. (**b**) The enlarged section of the vapor pressure data between 400 K and 1600 K shows the good fit with the equation.

To test the results of our approach, independent data (other than the handbook [12]) were also used to examine the accuracy of the relations provided by GA. It is seen that for element Li, the results from GA not only follow the same trend as that reported by Kondo et al. [22], but it can also provide data up to a much higher temperature (Figure 6) with a mean absolute error of 1.25 × 10^−2^ atm.

### 3.3. Quantification of Uncertainty and Reliability of Our Results

Pressure predicted using the coefficients provided by GA is compared with the experimental value. The uncertainty in prediction is represented using the following relation:

(7) $$U=\frac{\left(P_{cal}-P_{exp}\right)}{P_{exp}}\times 100\;$$

**Figure 7:**
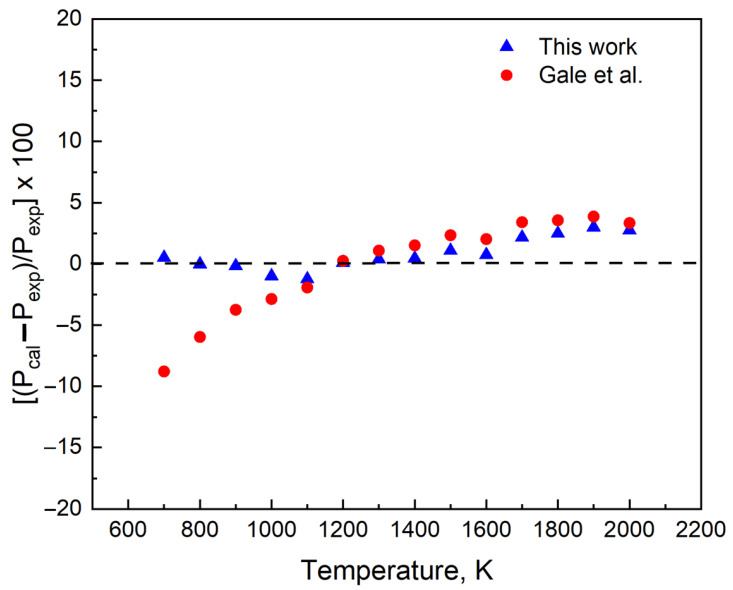
The differences in the vapor pressure data of the recommended relation and that using the previous relation Gale et al. [14] from the experimental data of Hultgren et al. [12] for Pb.

$P_{cal}$ is the pressure predicted using the coefficients A, B, C, and D in Equation (2) and $P_{exp}$ is the experimental pressure collected from [12]. Using element Pb as an example (Figure 7), we find that the pressure predicted ($P_{cal}$) is within 3% of the experimental value. 

**Figure 8:**
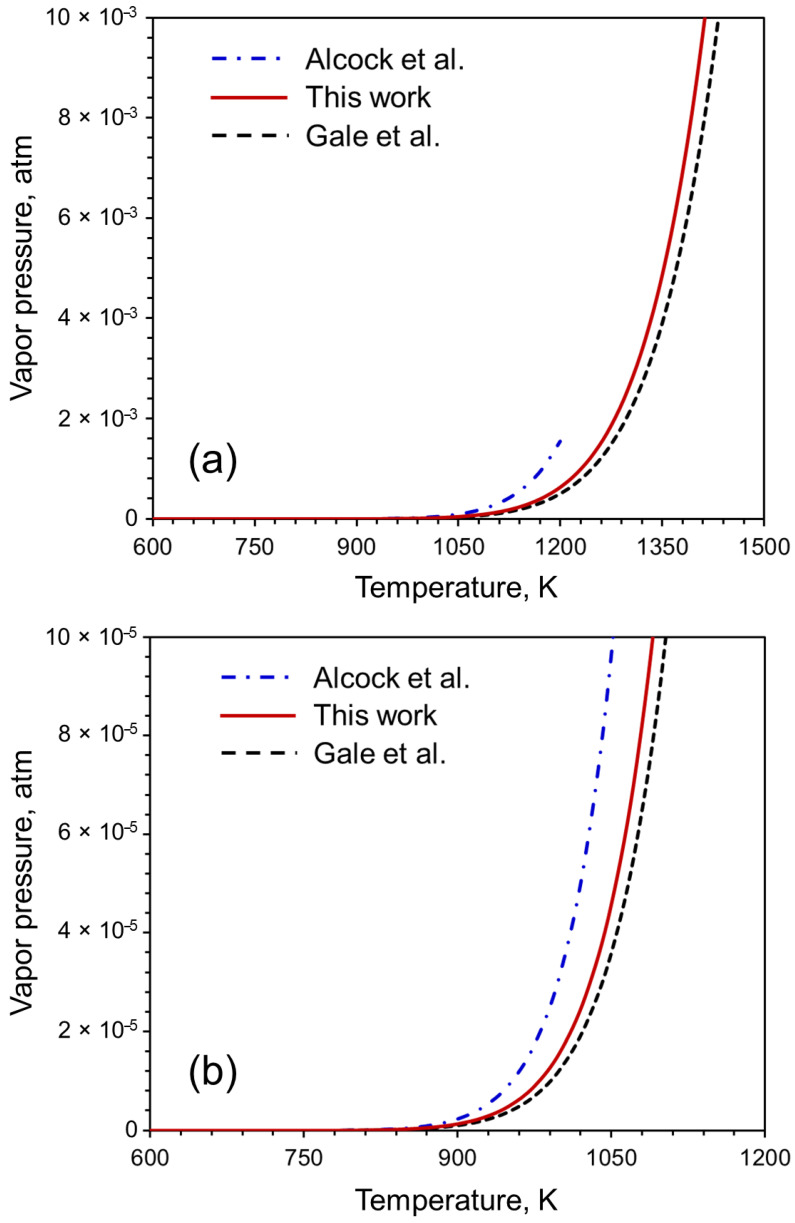
(**a**) Evaluation of reliability of the proposed equation for calculating vapor pressure. Here, we consider Pb as an example for which data are available between 600 K and 1200 K in works of Alcock et al. [13] and Gale et al. [14] in the range 600 K to 2030 K. (**b**) A zoomed in version of figure (**a**) within the temperature range of 600 K to 1200 K and between 0 atm and 1 × 10^−4^ atm vapor pressure.

The reliability of our proposed equation of vapor pressure can be evaluated by comparing the vapor pressure values computed using our equation with the vapor pressure values in the literature. The calculated values of the vapor pressure of Pb (Figure 8) are compared with those computed using the coefficients provided by Alcock et al. [13] and Gale et al. [14]. The data are available between 600 K and 1200 K in Alcock et al. [13]. and from 600 K to 2030 K in Gale et al. [14]. The coefficients are valid between 600 K and 2600 K. Figure 8 shows that our data is within the range of the data available in the literature. Therefore, our data is reliable as well as covers a wider range of temperatures that is not currently available in the literature.

### 3.4. Sources of Error

GA is a robust tool to fit non-linear, non-differentiable functions, and the accuracy of the fit can depend on various factors such as the number of generations, initial population size, cross-over ratio, and mutation factor. This approach of data fitting using GA may contribute to some errors. We were able to minimize the error from GA by choosing a large number of generations as 50,000. In addition, it is evident from Figure 4 that the fitness error reaches a low value of 0.002 atm at the end of the calculations ensuring a good fit.

Since both experimental data and data from the Clausius Clapeyron relation are used as inputs in GA, incorrect experimental data can also result in errors. Often the experiments for vapor pressure data were not available for high-purity elements. For example, vapor pressure measurements are available for commercially pure elements which often contain impurities. The presence of a substantial level of impurity in the element of interest indicates that the measured vapor pressure may not reflect the correct vapor pressure of the element unless they are corrected [23].

## 4. Summary and Conclusions

We synthesize vapor pressure data from the literature and use the Clausius Clapeyron relation to provide the vapor pressure versus temperature relations for fifty elements. The relations are applicable for a wide range of temperatures and provide vapor pressure from 10^−8^ atm (1.013 × 10^−3^ Pa) to 10 atm (1.013 × 10^6^ Pa) with a very low root mean square error in the order of 10^−2^ atm. We found that the vapor pressure values computed using the relations are consistent with the independent experimental data. In addition, the relations are capable of predicting the boiling points of elements accurately. Finally, the relations are found to be reliable in predicting the vapor pressure with a maximum deviation of 10^−3^ atm pressure from the existing database. 

## Figures and Tables

**Table 1:** Boiling points and enthalpy of vaporization of elements used in the Clausius Clapeyron equation [20,21].

Element	Boiling Point (K)	Enthalpy of Vaporization (kJ/mol)
Ag	2483	254
Al	2743	284
Au	3243	342
B	4203	508
Bi	1833	179
Ca	1760	153
Cd	1038	100
Ce	3743	398
Co	3173	390
Cr	2945	347
Cs	963.2	66.1
Cu	2868	305
Fe	3134	354
Ga	2673	256
Ge	3103	330
Hf	4876	648
In	2273	225
K	1047	79.1
La	3743	400
Li	1603	136
Lu	3603	414
Mg	1383	132
Mn	2373	225
Mo	4885	617
Na	1163	97.4
Nb	5017	694
Nd	3303	289
Ni	3003	379
Os	5273	678
Pb	2017	177
Pd	3233	380
Pt	4100	510
Rb	961.2	69
Re	5903	707
Rh	4000	531
Sc	3003	310
Se	958	95.5
Si	3533	383
Sm	2173	192
Sn	2893	290
Sr	1653	141
Ta	5693	753
Te	1263	114
Ti	3533	427
Tl	1733	162
V	3680	444
W	6203	774
Y	3203	390
Zn	1180	115
Zr	4650	591

Note: The data for Fe were taken from reference 21, while for rest all elements data were taken from ref. [20].

**Table 2:** Recommended coefficients for the vapor pressure of elements expressed by $logP=-\frac{A}{T}+B+C\;logT+10^{-3}DT$ where *P* is pressure in atm and *T* is the temperature in K.

Element	*A*	*B*	*C*	*D*	Temperature Range (K)	RMSE
Ag	21,330	65.78	−18.16	1.8	1100 to 3050	0.051
Al	12,210	−27.06	10.09	−1.16	1200 to 3370	0.062
Au	29,920	85.62	−23.53	1.913	1400 to 3975	0.100
B	31,710	22.78	−4.39	0.1608	2000 to 5000	0.001
Bi	10,430	10.7	−1.582	0.079	800 to 2280	0.060
Ca	11,610	34.36	−9.137	1.076	700 to 2255	0.024
Cd	6994	28.33	−7.699	1.57	420 to1300	0.016
Ce	22,390	9.125	−0.869	−0.010	1600 to 4575	0.039
Co	25,540	35.6	−8.461	0.652	1500 to 3750	0.043
Cr	21,790	15.86	−2.420	−0.024	1400 to 3525	0.010
Cs	4393	15.66	−3.973	0.782	400 to 1340	0.032
Cu	21,650	46.72	−12.26	1.124	1200 to 3500	0.105
Fe	27,180	50.1	−12.62	0.8586	1400 to 3775	0.003
Ga	25,040	96.49	−27.48	2.637	1050 to 3350	0.330
Ge	82,050	386.3	−110.7	8.599	1500 to 3750	0.370
Hf	45,980	84.44	−22.19	1.402	2200 to 5675	0.093
In	6714	−44.24	15.23	−1.726	1000 to 2790	0.365
K	4941	12.69	−2.79	0.436	400 to 1410	0.021
La	21,470	2.473	1.067	−0.147	1600 to 4575	0.010
Li	6416	−17.58	7.536	−1.604	700 to 2075	0.087
Lu	29,330	58.79	−15.47	1.214	1600 to 4325	0.054
Mg	12,040	67.15	−20.14	3.482	600 to 1730	0.035
Mn	23,600	85.49	−23.92	2.191	1000 to 3000	0.118
Mo	40,260	43.96	−10.43	0.565	2200 to 5760	0.022
Na	5764	11.19	−2.152	0.316	500 to1510	0.023
Nb	45,520	48.26	−11.41	0.606	2400 to 5800	0.051
Nd	18,880	25.2	−5.937	0.427	1290 to 4225	0.074
Ni	−4552	−165.9	51.135	−4.476	1500 to 3525	0.055
Os	34,690	−21.13	8.276	−0.587	2600 to 6200	0.092
Pb	9985	7.673	−0.834	0.016	800 to 2600	0.009
Pd	25,800	55.09	−14.655	1.337	1400 to 3875	0.028
Pt	31,660	24.88	−5.016	0.235	1900 to 4850	0.001
Rb	3735	−2.693	2.567	−1.123	400 to1325	0.035
Re	50,300	52.63	−12.51	0.521	2800 to 7025	0.052
Rh	26,670	2.401	1.319	−0.119	2000 to 4720	0.199
Sc	16,750	−12.21	5.808	−0.802	1400 to 3700	0.121
Se	6532	24.87	−6.464	1.272	500 to 1190	0.003
Si	17,250	−15.97	6.403	−0.5281	1700 to 4300	0.064
Sm	19,140	91.49	−26.8	3.113	800 to 2800	0.289
Sn	15,900	7.795	−0.674	0.012	1200 to 3600	0.014
Sr	9654	23.6	−5.883	0.711	830 to 2125	0.010
Ta	47,320	34.75	−7.534	0.326	2800 to 6650	0.066
Te	12,440	73.85	−22.01	3.371	600 to 1625	0.349
Ti	26,910	28.53	−6.305	0.413	1600 to 4190	0.080
Tl	8591	−0.38	1.895	−0.461	700 to 2200	0.012
V	37,240	73.27	−18.97	1.221	1800 to 4375	0.160
W	83,040	151.1	−38.85	1.551	3000 to7325	0.262
Y	−18,360	−246.3	74.075	−5.968	1500 to 3800	0.171
Zn	8681	36.95	−10.36	1.888	500 to1475	0.020
Zr	28,580	−0.651	1.95	−0.076	2200 to 5475	0.031

## Data Availability

Not applicable.

## Appendix A

Table A1 indicates the difference between the experimental data [12] and the fitting results in Figure 2 between 1700 K and 3400 K. The coefficients *A* = 17,250, *B* = −15.97, *C* = 6.403 and *D* = −0.5281 shown in Table 2 are used in Equation (2) to generate the fitting results. A good agreement between the experimental data and the fitting results is observed.

**Table A1:** Comparison between the experimental data [12] and the fitting results in Figure 2 between 1700 K and 3400 K.

Temperature, K	Experimentally Measured Vapor Pressure, Atm	Vapor Pressure from the Fitted Equation, Atm
1700	4.5 × 10^−7^	4.67 × 10^−7^
1800	2.15 × 10^−6^	2.19 × 10^−6^
1900	8.74 × 10^−6^	8.74 × 10^−6^
2000	3.07 × 10^−5^	3.06 × 10^−5^
2200	2.7 × 10^−4^	2.68 × 10^−4^
2400	0.00164	0.00165
2600	0.00752	0.00773
2800	0.0278	0.02902
3000	0.0862	0.09114
3200	0.231	0.24713
3400	0.552	0.59291
